# Cellular reprogramming for pancreatic β-cell regeneration: clinical potential of small molecule control

**DOI:** 10.1186/2001-1326-3-6

**Published:** 2014-03-27

**Authors:** Ganesh N Pandian, Junichi Taniguchi, Hiroshi Sugiyama

**Affiliations:** 1 Institute for Integrated Cell-Material Sciences (iCeMS), Kyoto University, Sakyo, Kyoto 606-8502, Japan; 2 Department of Chemistry, Graduate School of Science, Kyoto University, Sakyo, Kyoto 606-8501, Japan

**Keywords:** Cellular reprogramming, Diabetes mellitus, Transcription factors Pancreatic β cells, Small molecules

## Abstract

Recent scientific breakthroughs in stem cell biology suggest that a sustainable treatment approach to cure diabetes mellitus (DM) can be achieved in the near future. However, the transplantation complexities and the difficulty in obtaining the stem cells from adult cells of pancreas, liver, bone morrow and other cells is a major concern. The epoch-making strategy of transcription-factor based cellular reprogramming suggest that these barriers could be overcome, and it is possible to reprogram any cells into functional β cells. Contemporary biological and analytical techniques help us to predict the key transcription factors needed for β-cell regeneration. These β cell-specific transcription factors could be modulated with diverse reprogramming protocols. Among cellular reprogramming strategies, small molecule approach gets proclaimed to have better clinical prospects because it does not involve genetic manipulation. Several small molecules targeting certain epigenetic enzymes and/or signaling pathways have been successful in helping to induce pancreatic β-cell specification. Recently, a synthetic DNA-based small molecule triggered targeted transcriptional activation of pancreas-related genes to suggest the possibility of achieving desired cellular phenotype in a precise mode. Here, we give a brief overview of treating DM by regenerating pancreatic β-cells from various cell sources. Through a comprehensive overview of the available transcription factors, small molecules and reprogramming strategies available for pancreatic β-cell regeneration, this review compiles the current progress made towards the generation of clinically relevant insulin-producing β-cells.

## Introduction

Diabetes mellitus (DM) is an endocrine disorder associated with hyperglycemia and results in severe damage to the blood vessels, eyes, kidneys and heart. According to the latest survey, 347 million people worldwide suffer from DM, and about 10% of adults in upper-middle- and middle-income countries have the highest prevalence of this disorder [1]. The DM pandemic could grow exponentially in the next few decades and hence; there is a huge economic burden on governments and individuals [2]. This chronic disease is categorized into three major types. Type I DM (T1DM) is an autoimmune disease characterized by insulin secretion deficiency instigated by the destruction of insulin-producing β cells [3]. The non-insulin-dependent type 2 DM (T2DM) is a metabolic disease identified by insulin resistance and pancreatic β cell dysfunction and is predominantly caused by a poor lifestyle [4]. Recent studies indicate that mutations in certain genes such as *MADD (IG20)* also play a critical role in causing T2DM [5]. Gestational DM is another major form of DM affecting about 3–10% of pregnancies, which in severe cases can lead to neonatal and intrauterine fetal mortality [6].

**Figure 1:**
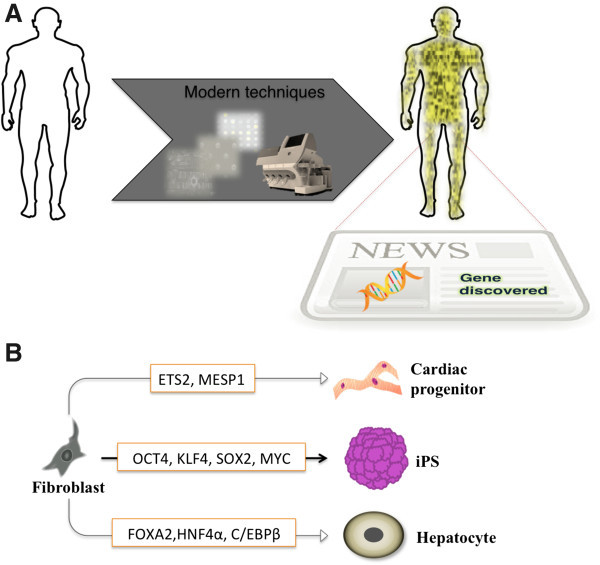
**Transcription factor-based cellular reprogramming. (A)** Modern experimental techniques like DNA chips, expression arrays and next generation sequencer (Shown in the arrow) facilitate us to gain insight into the human genome and identify novel genes/factors conferring to diseases and/or cell fate modulation **(B)** Enforced transcriptional activation of defined factors reprogram human somatic fibroblasts into different cell types like pluripotent stem cells [8], cardiac progenitors [9] and hepatocytes [10].

Functional β cells could be derived from human pancreatic stem/progenitor cells through differentiation protocols. However, resourcing issues and the lack for characterized markers hamper the employment of these cells. Induced pluripotent stem (iPS) cell technology extends the possibility of generating safe and functional pancreatic β cells without the possible risk of implant rejection and offers a potential cure for both T1DM and T2DM [7]. Recent progress in functional genomics provides us the sequence of 3 billion base pair human genome, and through loss-of-function studies we can identify cell fate modulating transcription factors (Figure 1A). Enforced transcriptional activation of some of these key genes can de-differentiate and/or trans-differentiate the human somatic cells like fibroblasts into different cell types (Figure 1B) [8-10].

It is now possible to reprogram majority cell type precisely across lineage boundaries into desired cell type including pancreatic β cells. Contemporary high-throughput and characterization studies facilitate the screening and identification of small molecules capable of modulating several such key transcription factors [11]. A novel DNA-based targeting epigenetic switch induced key transcription factors associated with insulin secretion [12]. In this review, we provide a critical overview of the strategies available for pancreatic β cell regeneration and list some of the well-known and recently identified transcription factors. We also give a detailed overview of the available reprogramming strategies including small-molecule control of cell fate, discuss the major barriers hindering their clinical use, and suggest future directions to achieve functional pancreatic β cells efficiently and safely.

## Review

### Treatment options for DM

Since the discovery of insulin in 1921, insulin replacement has become the main treatment for controlling plasma glucose level [13]. Various treatment options are now available to manage both T1DM and T2DM, and they rely largely on lifestyle changes such as dietary restrictions. The major drugs to treat DM include insulin, glucagon-like peptide 1 agonists, sulfonylureas, metformin, thiazolidinediones, α-glucosidase inhibitors, and dipeptidyl peptidase-4 inhibitors [14,15]. Despite remarkable progress and exciting discoveries over the past decade, a permanent cure for DM is yet to be achieved. The continuous need for antidiabetic drugs in DM treatment and chronic hyperglycemia lead to infections, ketoacidosis, hypoglycemia, and micro- and macrovascular disorders affecting the retina and nervous, renal, cardiovascular, and cerebrovascular systems [13]. It is also difficult to maintain long-term glycemic control in patients with DM [16,17].

Through innovative integration of a continuous glucose monitoring device and an insulin pump, a recent FDA-approved device called a bio artificial pancreas from Medtronic has been shown to improve the insulin treatment in T1DM [18]. Bio artificial pancreas technology is still at an early stage, and any long-term effects are yet to be evaluated. Organ replacement therapies such as pancreatic transplantation are other strategies available to treat DM; however, they have postoperative complications. Islet allograft transplantation to replace β cells is another minimally invasive strategy. However, these cell transplantation strategies rely largely on cadavers as donors [19]. Moreover, limitations such as toxicity arising from the prolonged use of immunosuppressants, graft cell loss and the damage caused by autoimmune responses are some major concerns [16,20]. The rapid advances in stem cell technologies suggest the possibility to overcome the abovementioned limitations of cell replacement therapy.

### Stem cell therapy for DM

Stem cells are characterized by their remarkable ability to self-renew and to acquire varying degrees of potency for differentiation. Depending on the kind of division (symmetric or asymmetric) modulated by the microenvironment, stem cells give rise to either an offspring preserving the characteristics of the parent stem cell or another progeny with different potency and lineage potential [13]. Throughout life, stem cells are maintained as tissue-specific adult stem cells in the body. Some of the primary stem cell sources available to achieve β cell replacement include pancreatic stem cells, hepatic stem cells and mesenchymal stem cells (MSCs). However, lack of characterization studies hampers the classification of any group of these cells as pancreatic stem cells. A group of cells classed as pancreatic duodenal homeobox 1 (PDX1)^+^/ insulin (INS)^+^/glucose transporter 2 (SLC2A2)^−^ cells isolated from the pancreatic duct and pancreatic islet tissues of transgenic mice have been classified as pancreatic progenitor cells [20]. Recently, Lima *et al.* reprogrammed the human exocrine pancreatic tissue into functional insulin producing β-like cells by suppressing epithelial-to-mesenchymal transition [21].

**Figure 2:**
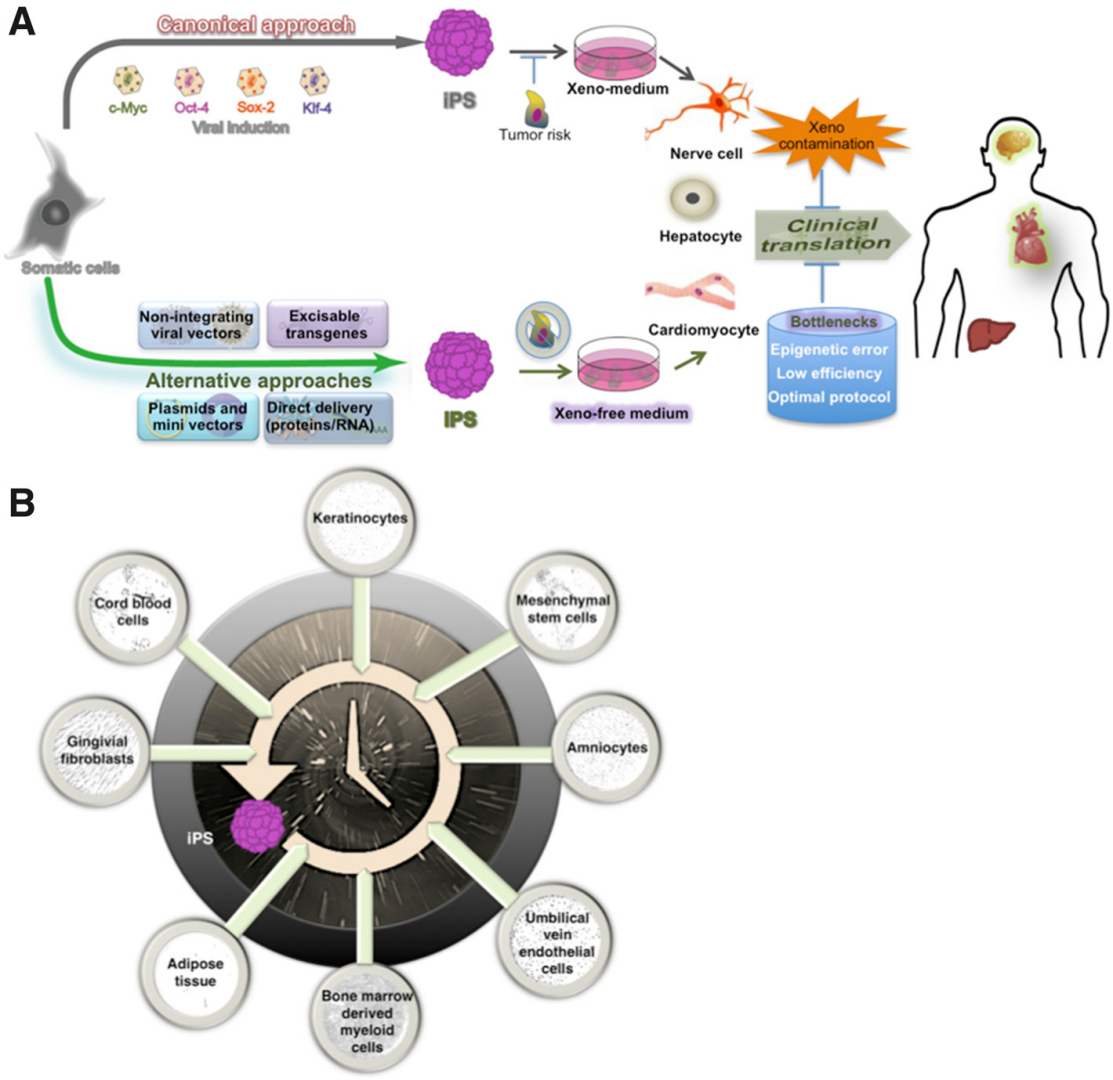
**Approaches to iPS cell generation and the obstacles to their clinical translation. (A)** In canonical approach (dark-gray arrow), retroviral derived iPS cells could differentiate into varied cell types. However, the risk of tumor formation and their culture in xeno-medium can inhibit the clinical translation of these cells. Alternative non-integrating approaches [30,31] in which the overexpression of defined reprograming factors in various ways (green arrowhead) generate iPS cells that circumvent tumor formation, and when cultured in xeno-free medium, can avoid the xeno contamination that hinders their clinical translation. Several bottlenecks, including epigenetic errors, low efficiency, and protocol optimization, are highlighted (blue cylinder). **(B)** Various cell sources (microscopic images) can be reset for a journey back in time to iPS cells (illustrated as the time machine icon) [30].

Considering the difficulty in obtaining pancreatic stem cells, bone marrow stem cells such as MSCs are preferred as they can be obtained easily [22]. The mechanism behind the MSC-mediated stimulation of β cell regeneration is still under debate [23]. Nevertheless, the evidence thus far clearly substantiates MSCs as a clinical-friendly cell source to treat DM owing to their immunomodulatory effects [24,25]. The shared developmental origin of hepatic, pancreatic and common progenitor cells suggests hepatic stem cells to be a better choice for achieving functional insulin-producing β cells [26]. Accordingly, enforced transcriptional activation of *Pdx1* and *Ngn3* by adenoviral transfection maintained blood glucose levels by inducing the differentiation of pancreatic endocrine and exocrine cells [27]. Embryonic stem cells (ESCs) are pluripotent in nature and might be stimulated to differentiate into pancreatic β cells through *in vitro* differentiation protocols. Human ESC-derived pancreatic progenitor cells had the capability to differentiate and mature, and they synthesized insulin after being transplanted into mice [28]. Recent reports on human ESC-derived insulin-producing cells and demonstration of their capability to treat artificially induced hyperglycemia in mice substantiate their therapeutic prospects [29]. However, the sourcing of ESCs and the ethical controversy regarding the destruction of human embryos to produce them is a major concern. The epoch-making discovery of iPS cell technology not only eased the ethical controversy associated with human ESC research but also opened up the possibility of reprogramming any cell to a desired cellular phenotype [30]. Artificial induction of pluripotency in human somatic cells through enforced transcriptional activation of four factors (OCT4, SOX2, c-MYC, and KLF4) has opened up new opportunities to reverse the fate of any terminally differentiated cells into pluripotent stem cells [31]. Several strategies are now available to achieve pluripotent stem cells (Figure 2A) from a variety of cell sources (Figure 2B) [30].

Because the resulting iPS cells have all the potential of ESCs [8], they can be readily differentiated into cells across lineage boundaries. Derivation of β cells from iPS cells can lead to clinically safe autologous cell populations and hence can be an ideal source for transplantation therapy. Using human iPS cells, Tateishi *et al.* first derived functional insulin-producing β cells forming islet-like clusters composed of C-peptide- and glucagon-positive cells [32]. In 2009, Maehr *et al.* demonstrated that iPS cells derived from the skin fibroblasts of a patient with T1DM could differentiate into insulin-producing/glucose-responsive cells [33]. This innovative approach not only overcame the barrier of immune rejection but also could aid in identifying the genomic aberrations associated with T1DM. These patient-derived iPS cells (termed DiPS) expressed and stained positive for some of the pancreatic endocrine markers including insulin, PDX1, NKX2-2, glucagon and somatostatin. Interestingly, DiPS secreted insulin upon glucose stimulation in a dose-dependent manner.

Zhang *et al.* improved the efficiency of differentiation protocols and generated mature β-like cells expressing markers (C-peptide, PDX1, GLUT2, MAFA, NKX6-1, ISL-1 and NEUROD1) similar to adult β cells [34]. In 2010, Alipio *et al.* demonstrated the clinical potential of iPS-cell-derived β cells by transplanting them into a mouse model of T2DM. The transplanted cells corrected the blood glucose levels and hyperglycemia for a long time concomitantly with an increase in the *in vivo* concentration of insulin [35]. It is important to note here that the iPS-derived insulin-producing cells engrafted stably and became distributed to survive within the host tissue. This approach was also successful in a mouse model of T1DM and hence, suggested the clinical potential of iPS-derived insulin-producing cells. Thus, it is now possible to generate human pancreatic endoderm and differentiate them *in vivo* into glucose responsive mature islet cell types capable of secreting C-peptide, insulin, glucagon, or somatostatin. However, the current differentiation protocols could not result in the generation of clinically significant levels of human endocrine hormone. Recent reports focus on the importance of deriving pancreatic progenitor cells that follow *in vivo* pancreatic developmental ontogeny during differentiation of pluripotent stem cells [36,37]. Evaluation of long-term complications and identification of key transcription factors could further support the therapeutic potential of iPS cells in treating patients with DM.

## Cellular reprogramming and beta cell regeneration

### Transcription factors and pancreatic development

There has been an exponential increase in studies reporting the identification of key genes/factors capable of switching the cell fate from one state to another. Several transcription factors operating at various stages of development govern the sequential cascade of transcriptional ‘ON’ and ‘OFF’ mechanisms orchestrating pancreatic organogenesis. Through functional genomics and genetic studies in mice, the functional role of some transcription factors in coordinating normal organogenesis and the subsequent specification into distinctive endocrine cell types got identified [38]. Interestingly, single nucleotide polymorphisms occur frequently in the binding sites of these transcription factors [39]. Pancreatic and duodenal homeobox 1 (PDX1) play a vital role as the master regulator in different stages of pancreatic development and islet cell morphogenesis [40]. PDX1 coordinates the intricate transcriptional regulatory interactions between the transcription factors, which confers to the multi-potent pancreatic progenitors [38]. Considering its association in regulating key β cell genes, PDX1 could be a pharmacological target for β-cell defects in T2DM [40]. Loss-of-function studies suggested the key role of the self-regulating pancreas transcription factor 1 subunit (PTF1A) in ensuring the lineage commitment of pancreatic buds towards pancreatic progenitor fate [41]. The role of GATA factors in pancreas development got verified in a mouse model where the simultaneous inactivation of both *Gata4* and *Gata6* perturbed the proliferation of pancreatic progenitor cells and caused faulty branching morphogenesis [42]. Likewise, simultaneous removal of *Foxa1* and *Foxa2* disturbed the early event regulating the pancreas formation [43]. Interestingly, in both the above-mentioned studies, loss of *Pdx1* expression got associated with pancreatic hypoplasia [42,43]. The sequential activation of the hepatocyte nuclear factors (HNFs) like HNF1β and HNF6 expressing in endoderm and Pdx1 modulate the generation of pancreatic progenitors [44]. Lynn *et al.* showed that the Sry/HMG box transcription factor SOX9 could coordinate transcription factors expressed in pancreatic progenitor cells [45].

The NK-homeodomain factor *Nkx2.2* and its downstream gene *Nkx6.1* plays a major role in the development of insulin producing β-cells [46]. The LIM homeodomain protein ISL1 is a critical factor for the development of the dorsal exocrine pancreas and could direct mesenchymal cells into either an endocrine or an exocrine state [47]. Itkin-Ansari *et al.* demonstrated that the basic helix-loop-helix transcription factor NeuroD1 functions as both a transcriptional activator and repressor in the establishment and maintenance of mature endocrine cells [48]. Pax4 operate in concert with Pax6 to regulate normal pancreatic endocrine development and the master regulator Pdx-1 controls the transcription of the insulin, GLUT-2, GK, and Nkx6-1 in adult β-cells [49]. The transcription factor neurogenin 3 (Ngn3) regulate the expression of Isl1, Pax4, Pax6, and NeuroD1 and trigger the differentiation of the β-cells from pancreatic endoderm to specify four endocrine cell lineages of the pancreas [50]. Smith *et al*. showed that the coordinating role of the regulatory factor X, 6 (Rfx6) in the completion of the differentiation process initiated by Ngn3. Since Rfx6 lies downstream of Ngn3 and upstream of other islet transcription factors, a comprehensive understanding of the role of Rfx6 could aid the generation of β-cells from patients with DM [51]. The essential role of the Maf factors like MafB and MafA in the pancreatic β-cell differentiation and maturation is known [52]. Gaining insights into the binding sites of diverse transcription factors could aid the uncovering of underexplored regulatory pathways affecting pancreatic β cells and causing DM.

### Transcription factor-based cellular reprogramming

**Figure 3:**
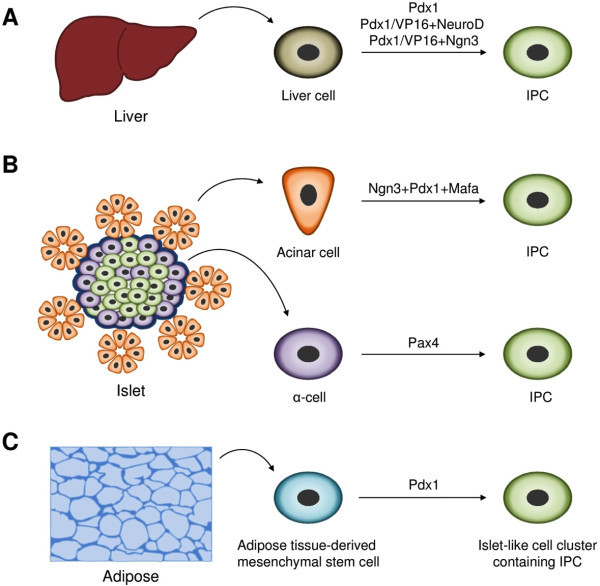
**Insulin producing cells (IPCs) from various cell sources. (A)** Overexpression of the defined exogenous transcription factors (Pdx1; Pdx1/VP16 (fusion protein) + NeuroD; Pdx1/VP16 (fusion protein) + Ngn3) in liver cells generate IPCs [53,54]. **(B)** IPCs could also be induced from pancreatic non-β cells such as acinar cells and α-cells by forced expression of Ngn3 + Pdx1 + Mafa and Pax4, respectively [58]. **(C)** Adipose tissue-derived stem cells could be differentiated into functional IPCs by transducing Pdx1 [59].

Cellular reprogramming strategies also suggest the possibility to achieve insulin-producing β cells by trans-differentiating a wide range of cells. In particular, the cells like hepatocytes and non-endocrine pancreatic cells sharing the common lineage have a better chance to get reprogrammed into β cells. Although these cells cannot secrete insulin owing to the absence of some key factors, they express some common proteins. For example, hepatocytes cannot make insulin from proinsulin but express the factors responsible for stimulus-secretion coupling like glucose transporter 2 and glucokinase, which also occur in the β cell. Hence, fewer factors could be required to reprogram such cells into insulin producing β cells. Accordingly, adenovirus-mediated gene transfer of Pdx1 into mice reprogrammed hepatocytes into cells capable of decreasing blood-glucose levels by producing insulin [53] and when fused to VP16 transcriptional activation domain (Pdx1/VP16), the efficiency of insulin producing cell generation were significantly enhanced (Figure 3A) [54]. Subsequently, the adenoviral transfection of PDX1 reprogrammed hepatocytes or the cells from human liver biopsies into cells capable of controlling blood glucose levels in DM animals (Figure 3A) [55]. Using the adeno-associated virus-mediated transfer, Wang *et al.* later demonstrated that the adenovirus transduction-triggered immune response play a critical role in the reprogramming of hepatocytes into β-like cells [56]. Likewise, viral-mediated delivery of Ngn3, MafA, and Pdx1 reprogrammed the acinar cells of the immunodeficient mice into insulin-positive cells (Figure 3B) [57]. Despite the problems like the inability to form organized and fully functional islet structures, this proof-of-concept study suggested the possibility to transform nonendocrine pancreatic cells into cells having the β-like function.

Through the ectopic expression of Pax4, Collombat *et al.* reprogrammed pancreatic progenitor cells expressing Pdx1 into glucagon expressing α cells, which then got reprogrammed into insulin-positive cells (Figure 3B) [58]. However, in the older animals, the normoglycemia could not be restored and the reason behind this effect is unclear. Nevertheless, this study suggested that to achieve glucose-responsive, insulin-secreting β cells, alpha cells from DM patients could serve as a potential cell source. Kajiyama *et al*. showed that, upon Pdx1 transfection, the adipose tissue-derived stem cells could differentiate into IPCs in vivo and alleviate hyperglycemia in diabetic mice (Figure 3C) [59]. Thus, the identification of optimal cell sources and development of clinical-friendly reprogramming approaches could lead to long-term stable treatment strategy to cure DM.

### Small molecules for pancreatic β cell regeneration

Recently, Deng *et al.* showed that it is possible to artificially induce pluripotency in mouse somatic cells using seven small molecules alone [60]. This transgene free cellular reprogramming strategy is expected to be clinically relevant as small molecules are mostly non immunogenic and their mode of employment is relatively easier than the conventional reprogramming approaches [61]. Several small molecules like the inhibitors of epigenetic enzymes and signalling pathway factors aided the generation of pluripotent stem cells from both mouse and human somatic cells [29]. Some of the small molecules that activate or inhibit certain factors could also promote the differentiation of pluripotent stem cells and even somatic cells into functional β cells. Chen *et al.* identified through high-content chemical screening that a small molecule called (−)-indolactam V could trigger the differentiation of human ESCs into Pdx1-expressing cells [62]. In a subsequent study, the TGF-β signalling pathway activating small molecules induced endoderm formation from mouse ESCs [63]. This inducer of definitive endoderm (IDE)-1 and its derivative (IDE-2) further enabled the induction of pancreatic progenitors from mouse ESCs when treated along with (−)-indolactam V [63]. The versatile differentiating agent retinoic acid enhanced the generation of homogenous pancreatic PDX1 (+) pancreatic progenitor cells from human ESCs and also promoted further differentiation into β cells [64,65]. Dadheech *et al.* recently isolated a small molecule called Swerstin from a perennial herb called *Enicostemma littorale* and demonstrated its ability to differentiate NIH3T3 cells into islet-like clusters [66]. Furthermore, they showed that normoglycemia could be restored upon the transplantation of these cells in diabetic Balb/c mice. Using high-throughput and cell-based screening studies, Shen *et al.* identified and characterized a novel small molecule ‘WS6’ having the capability to promote β cell proliferation in the primary islets of both rodent and human [67]. Expression of PDX1 plays an essential role in both β-cell regeneration and maturation. In a very recent study, Yuan *et al.* used a high-throughput qPCR study to screen 60,000 compounds and identified a small molecule capable of inducing PDX1 expression in the human PANC-1 ductal carcinoma cell line [68]. This novel small molecule called BRD7552 triggered transcriptionally permissive epigenetic changes in the PDX1 promoter region and has shown the capability to induce PDX1 expression in multiple cell lines and primary human cells. Likewise, some known epigenome modifying small molecules have shown the capability to promote β − cell differentiation by inducing key transcription factors. For example, the DNA methyl transferase inhibitor 5-aza-2′-deoxycytidine (5-AZA) triggered transcriptional activation of Ngn3 expression, which in turn promoted endocrine differentiation in the human PANC-1 pancreatic ductal cell line [69] (Table 1).

**Table 1:** Small molecules and pancreatic β-cell regeneration

**Chemical**	**Target**	**Source**	**Reference**	**Notes**
Indolactam V	Pancreas progenitor	Human/mouse definitive endoderm	[62]	PKC activator.

Induce definitive endoderm (IDE 1 (above) and IDE 2 (below)	Endoderm	Mouse ESCs	[63]	TGF-β activator. Enables induction of pancreatic progenitors from mESCs with Indolactam V

Retinoic acid (RA)	Pancreatic cells, β-cells	Definitive endoderm	[64,65]	

Swertisin	Islet-like cell clusters	Mouse NIH3T3 cells	[66]	

WS6	-	β-cells	[67]	IκB kinase pathway activator (Promotes proliferation of β cells)

BRD7552	-	Human islets and ductal cells	[68]	PDX1 expression.

5-aza-2’-deoxycytidine (5-AZA)	Pancreatic endocrine cells	PANC-1 human ductal cell line	[69]	DNA methylation inhibitor.
				

*Abbreviations, PKC* protein kinase C, *ESCs* embryonic stem cells, *TGF-β* transforming growth factor β.

**Figure 4:**
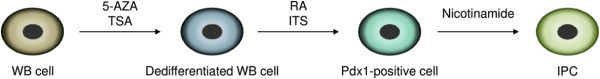
**Small molecules and β cell induction.** Liver epithelial stem-like white blood (WB) cells can be reprogrammed into insulin-producing cells (IPCs) by sequential protocol using small molecules [68]. In the first stage, WB cells are dedifferentiated with 5-aza-2’-deoxycytidine (5-AZA) and Trichostatin A (TSA). Then, retinoic acid (RA) and a mix of insulin, transferrin and selenite (ITS) are added to induce PDX1-positive pancreatic precursor cells. Nicotinamide promotes maturation of IPCs in the last stage.

Direct differentiation of hepatic stem-like WB cells into insulin-producing cells got achieved using a combination small molecules like selenite, 5-AZA, RA and Trichostatin A (TSA), a regulator of chromatin remodeling and the factors like insulin and transferrin [70] (Figure 4). This study suggests that it is possible to trans-differentiate optimal cell source like hepatocytes into functional β cells. However, lack of selectivity of some chromatin modifying enzymes and requirement of multiple factors is a major concern.

### Distinct DNA-based epigenetic switches for gene regulatory networks

**Figure 5:**
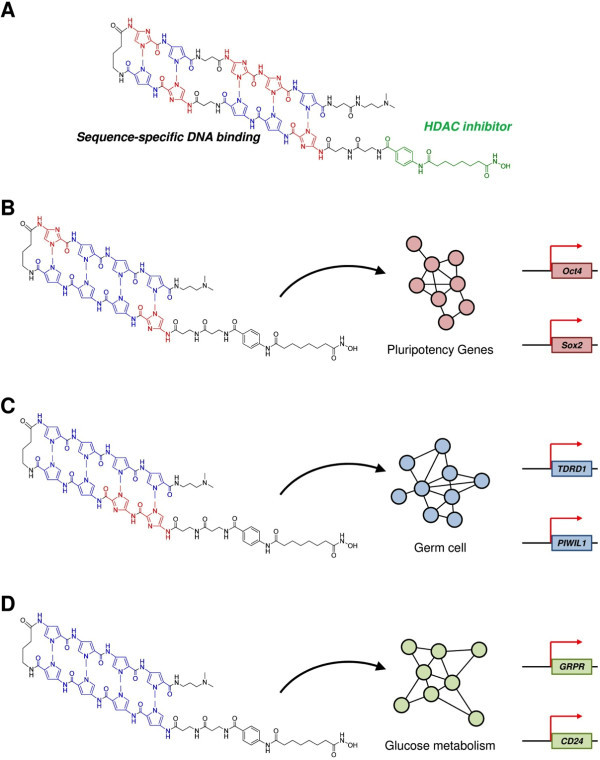
**SAHA-PIP: potential tool for selective transcriptional activation. (A)** Chemical structure of SAHA-pyrrole-imidazole polyamide (SAHA-PIP) conjugate capable of site-specific acetylation in the promoter region of p16 tumor suppressor gene [71]. **(B)** SAHA-PIP `**δ`** triggers the core pluripotency gene network in mouse embryonic fibroblasts through site-specific epigenetic activation. In human dermal fibroblasts [74], **(C)** SAHA-PIP **K** and **(D)** SAHA-PIP **A** activates gene regulatory network associated with and germ cell and pancreas function such as glucose metabolism, respectively [12,75].

In recent times, small molecules with versatile properties have shown the capability to induce multiple factors. SAHA-PIP is one such multi-target small molecule having sequence-specific DNA-binding hairpin pyrrole–imidazole polyamides (PIPs) and SAHA, a pan-HDAC inhibitor. Synthesized SAHA-PIPs targeting the promoter region of the tumour suppressor gene p16 triggered sequence-specific acetylation in HeLa cells (Figure 5A) [71]. Screening studies with a distinct set of SAHA-PIPs revealed certain SAHA-PIPs capable of differentially inducing pluripotency genes in mouse embryonic fibroblasts (MEFs) through activation of epigenetic marks associated with transcriptionally permissive chromatin [72,73].

Subsequent screening studies with a different set of SAHA-PIPs revealed a potent SAHA-PIP called **δ** capable of inducing multiple pluripotency genes and epithelial cell markers [74]. Since δ-OMe, the non-functional SAHA-PIP and SAHA alone did not activate any pluripotency genes; it is reasonable to assume the role of SAHA and PIP as the functional and DNA recognition module, respectively. A larger number of the **δ**-induced genes belonged to the intricate core pluripotency gene circuitry that includes 345 genes. Hence, PIP in **δ** could be directing SAHA to the usually silent core pluripotency gene network for site-specific epigenetic activation (Figure 5B).

Recently, a SAHA-PIP called **K** got categorized as the first-ever small molecule capable of enforcing transcriptional activation of meiosis-regulating germ cell genes in a human somatic cell [75]. This result substantiates the remarkable ability of SAHA-PIP to activate silent genes because these germ cell specific meiotic markers do not normally occur in a somatic cell. GRPR (gastrin-releasing peptide receptor) is a highly expressed factor in pancreas and assists insulin secretion by stimulating the autonomic nerves (Figure 5C) [76]. CD24 (cluster of differentiation 24) is a cell adhesion molecule and a recent study revealed CD24 as a marker for PDX1-positive pancreatic progenitors derived from human ESCs [77]. CD24-positive cells were shown to differentiate into insulin-producing cells. Recent screening studies with an available set of 32 SAHA-PIPs revealed that a SAHA-PIP called **A** (also labelled as **1**) could activate these two genes in human dermal fibroblasts. Moreover, transcriptome analysis suggested that effect of **A** is associated with pancreas-related function such as glucose metabolism and diabetes [10] (Figure 5D). Chromatin immune precipitation studies have revealed that SAHA-PIP can induce site-specific chromatin modification to activate the typically ‘silent genes’ in human somatic cells [75]. Because of the tunable potential, SAHA-PIP could be developed as a novel tool to achieve precisely reprogrammed pluripotent stem cell and/or insulin producing β cell. Precisely reprogrammed cells with fewer factors should have better clinical prospects and also could facilitate the achievement of homogenous insulin producing cells, which is a major bottleneck that hampers the clinical translation of artificially reprogrammed cells.

## Conclusion and Outlook

In general, diseases are characterized by dysregulation in the transcriptional machinery, which is responsible for the maintenance of the cellular homeostasis [78]. Recently, clinicians have turned their attention to genetic knowledge based therapeutic strategies to treat incurable diseases like DM [30]. Current gene expression profiling techniques helps us in predicting the transcription factors conferring to β cell regeneration [13]. In recent years, an increasing number of genes get classified as the potential therapeutic targets and/or cell-fate determining transcription factors [5,10]. With the rise of cellular reprogramming strategy, it is now possible to modulate these transcription factors and achieve desired cellular phenotype from a variety of cell sources. The reprogrammed insulin producing cells should be functionally matured for achieving clinically relevant numbers of endocrine cells. Moreover, these cells should also survive long and be engrafted properly inside the host cell environment. To achieve such complex feat, strategies to achieve identical islet-like structures having optimal access to nutrient and oxygen after engraftment *in vivo* is required.

Optimal cell sources and the reprogramming strategies also complicate the existing difficulties. For example, some of the currently available reprogramming protocols harbor the risk of transgene integration and mutagenesis, which are not clinical friendly [29]. Consequently, transgene free approaches to reprogram cells like employment of RNA, proteins and/or small molecules have been gaining attention. In particular, reprogramming cells with small molecules alone is accepted to have better clinical prospects than the other reprogramming strategies.

Small molecules are also the most preferred drugs among the clinicians and each year they out number the other biological drugs [79]. High-throughput studies revealed several small molecule inhibitors and/or activators that can aid the cellular reprogramming and even the generation of β cells. However, lack of sequence-specificity and the requirement of multiple small molecules necessitate the development of small molecules capable of mimicking the efficacy of their natural counterparts. To achieve the coordinated orchestration of gene expression observed in nature, small molecules should act at both genetic and epigenetic level to regulate the extremely complex gene regulatory networks. A promising way to achieve targeted transcriptional activation is to develop epigenetically active small molecules that could be preprogrammed to bind to specific DNA sequences. One such class of multi-target small molecules having the potential to achieve precise cellular reprogramming is the customizable synthetic SAHA-PIPs capable of modulating distinct biological networks (Figure 5). Synthetic PIPs gain advantages over other natural DNA-binding proteins as effective transcriptional activators because they possess flexible covalent sites and can bind to the methylated DNA sequences and disrupt the packed chromatin structure [80-82]. Modification in the structure of SAHA altered the specificity of `**δ** ` towards a specific HDAC enzyme [83]. Thus, it is possible to attach other epigenetic enzyme inhibitors to trigger variable effects. Ligands that recognize 15–16 base pairs can recognize a single site within the 3 billion base pair human genome [84]. Major regions of the iPS epigenome retain the epigenetic memory of their tissue of origin [85]. Chromatin-modifying enzymes act as both facilitators of and barriers to the epigenetic remodeling of differentiated cells into pluripotent stem cells [86]. Hence, selective chromatin modifiers could modulate the complicated transcriptional network, with less exogenous transcription factors, thus efficiently reprogramming somatic cells. In this context, small molecules, such as SAHA-PIPs, that can induce sequence-specific chromatin modifications may be developed to erase the epigenetic memory and aid the generation of clinical-grade reprogrammed cells [87,88]. Cellular reprogramming using small molecules alone also has certain limitations. For instance, a certain bioactive small molecule can have variable efficacy against different cell types. Cellular reprogramming with small molecules alone can also trigger genetic instability and the genetic integrity of the reprogrammed cells might get affected [61]. The remarkable ability of pluripotent stem cells to proliferate could also be an issue because the residual undifferentiated cells may cause tumor formation. Therefore, the clinical potential of the reprogrammed cells could be increased with the development of small molecules capable of selectively eliminating human pluripotent cells like PluriSIn#1 [89] and/or programmable SAHA-PIPs.

Identification of novel transcription factors and development of strategies for their modulation could lead to effective regeneration of pancreatic β-cells. Importantly, even after transplantation these precisely reprogrammed cells should still retain the capability to produce insulin. The safety requirements associated with any transplantation studies like dosage, genetic stability, possible pathogenicity and toxicity towards host tissue needs to be considered. Nevertheless, recent developments in diabetes research and increasing interest on the intellectual integration of diverse techniques suggest that a permanent cure is not too far.

## Competing interests

The authors have declared no conflict of interests.

## Authors’ contributions

The author GNP conceived the content of the manuscript with literature analyses and drafted the manuscript. HS provided intellectual comments and reorganized the content of the manuscript. JT collected literature and made figures. All authors read and approved the final manuscript.
